# A Review of the Effects of Stress on Dairy Cattle Behaviour

**DOI:** 10.3390/ani14142038

**Published:** 2024-07-11

**Authors:** Viktor Jurkovich, Péter Hejel, Levente Kovács

**Affiliations:** 1 Centre for Animal Welfare, University of Veterinary Medicine, István u. 2, H-1078 Budapest, Hungary; jurkovich.viktor@univet.hu; 2 Department of Animal Hygiene, Herd Health and Mobile Clinic, University of Veterinary Medicine, István u. 2, H-1078 Budapest, Hungary; hejel.peter@univet.hu; 3 Institute of Animal Sciences, Hungarian University of Agriculture and Life Sciences, Páter Károly u. 1, H-2100 Gödöllő, Hungary; 4 Bona Adventure Ltd., Peres u. 44, H-2100 Gödöllő, Hungary

**Keywords:** dairy cattle, stress, behaviour, coping

## Abstract

**Simple Summary:**

Today’s intensive dairy cattle farming causes more and more stress for the animals, which they must deal with in order to maintain a steady level of animal welfare and production. This review explores how stress affects behaviour in dairy cows, focusing on everyday stressors like pain, disease, heat, and social issues. It explains how these stressors can change how cows eat, interact, and feel. The review also discusses how cows adapt to stress physically and in their actions. It helps farmers better understand and manage cow stress, which is essential for cow welfare and farm productivity. Plus, it suggests areas for future research to learn more about how cows cope with stress.

**Abstract:**

In this narrative review, the authors summarise the relationship between stress and behaviour and how dairy cattle cope with stressors. Based on the available literature, the most common stressors in intensive dairy cattle farming, such as pain, disease, heat stress, poor comfort caused by technology, and social stress, are surveyed. The authors describe how these stressors modify the behaviour of dairy cattle, influencing their feeding patterns, social interactions, and overall well-being. Additionally, the review explores the effectiveness of various coping mechanisms employed by dairy cattle to mitigate stress, including physiological adaptations and behavioural responses. This review is a valuable resource for understanding and grading stress in dairy cattle through behavioural reactions. Elucidating the intricate interplay between stressors and behaviour offers insights into potential interventions to improve animal welfare and productivity in dairy farming. Furthermore, this review highlights areas for future research, suggesting avenues for more comprehensive behavioural studies to enhance our understanding of stress management strategies in dairy cattle.

## 1. The Behavioural Approach to Stress

As technology has evolved, dairy cattle housing has developed from full-time pasture-based systems to full-time housing [1]. In a human-built environment that provides everything necessary (feed, resting place, and social companions), cattle often face potentially provocative challenges that can cause stress [2]. Stress has been explained concerning Selye’s [3] concept as “exposure to nocuous environmental factors (stressors) elicits a nonspecific reaction”. This reaction (stress) can have detrimental effects in the form of changes in immune functions [4], hampered growth and reproduction [5,6,7,8], and even death by failure of adaptive mechanisms [9].

Exposure to various environmental events elicits physiological changes, described under two main headings for simplicity. (1) Cannon described the emergency reaction [10] and related it to the activation of the sympathetic–adrenal–medullary axis [11,12,13]. The emergency reaction is a short latency response involving hormonal factors (catecholamines) that enable the subject to mobilise its resources quickly for the metabolic requirements of fight or flight. (2) Selye initially described general adaptation syndrome [14] and characterised it by the release of adrenocorticotropic hormone from the anterior pituitary gland [15]. This reaction, in turn, activates the release of corticosteroids from the adrenal cortex. Corticosteroids amplify and extend the metabolic effects of catecholamines.

However, as a physiological mechanism, stress per se is not inherently wrong for the animals [9], as hormones released during stress periods are also part of the hormonal cascade in bovine parturition [16]. Glucocorticoids, particularly the steroid hormones cortisol and corticosterone, improve fitness by energy mobilisation in cattle [17,18] and may change behaviour during short-term stress [19,20,21]. However, severe chronic stress (prolonged periods of high cortisol concentrations) may decrease individual fitness by immunosuppression and atrophy of tissues [22,23]. There are also indications that stereotypies, such as non-nutritive oral behaviours, might be related to stress [24,25,26,27,28].

Knowledge of the behavioural characteristics of dairy cows is essential for the breeding, housing, and managing the animals. Behavioural and physiological differences between individuals in response to a stressor or an environmental challenge are often described with ‘coping style’ (the behavioural strategy used by the individual when faced with a stressor) [29,30,31] and ‘temperament’ [32,33,34]. In recent years, stress responsiveness has been associated with cattle behaviour, specifically temperament. The term ‘temperament’ is commonly used to describe the relatively stable differences in the behavioural predisposition of animals, which can be associated with psychobiological mechanisms [35]. According to Burrow [36], temperament is an animal’s behavioural response to human handling, while others defined this term as an animal’s leading personality or mood trait about humans [37]. Several definitions are given for coping styles, such as the behavioural and physiological efforts to master the situation [38,39].

Farm animals in intensive housing systems use strategies (escape, remove, search, wait) to cope with aversive situations. Regarding animal behaviour, research has focused on two distinct reaction patterns to stressful conditions. The first type is the active response, described initially by Cannon [10] as the ‘fight or flight’ response. This dynamic response is characterised by territorial control and aggression [40]. The second type is the conservation–withdrawal response [41], characterised by passive reactions such as immobility, urination, defecation, and low levels of aggression [42,43]. Based on a review of animal coping styles [39], it is preferable to use the terms proactive coping rather than active coping and reactive rather than passive coping (see more details in the next chapter).

Since the 1990s, several studies have focused on individual differences related to different coping strategies or differences in temperamental traits in dairy cattle in response to challenges using behavioural tests [42,44,45,46]. Extensive research has been conducted on farm animals using human exposure as a stressor to evaluate temperament and behavioural reactivity, mainly based on assessing the animal’s personal area [47]. The tests used for cattle were initially designed for laboratory animals. Besides the animals’ behaviour, stress affects many physiological systems controlled by the autonomic nervous system, including the cardiovascular system. Monitoring autonomic nervous system activity in dairy cattle has recently gained considerable interest worldwide [48]. Besides behavioural reactions, heart rate variability parameters, i.e., the short-term fluctuations in the variability of successive cardiac inter-beat intervals, were also helpful in differentiating between individual traits in lactating cows [33,45,49].

## 2. Purpose of Behavioural Responses to Stressors

The purpose of responses to any stressors is to cope with that stressor to keep allostasis, i.e., maintaining stability in changing conditions through physiological and behavioural events. Changes in body functions following exposure to a given stressor help the animal anticipate and respond to further challenges. This way, the animal can better adapt to its environment.

Stress responses relate to how animals perceive stressors and how they can respond to them. The conclusion of the classical experiment by Weiss et al. [50] was that the effects of a stressor depend more on whether the animal can predict or control it than on its physical characteristics. Control is generally exerted through performing an appropriate behaviour. Individuals often react differently to the same stressor under identical conditions. In another classical study, two different coping styles were observed in mice [51]. Aggressive individuals showed an active response to aversive situations. In a social setting, they react with flight or escape when defeated; in non-social cases, they actively avoid controllable shocks and sustained activity during an uncontrollable task. In contrast, non-aggressive individuals generally adopt a passive strategy. In social and non-social aversive situations, they reacted with immobility and withdrawal.

During proactive coping, the individual deals with challenges by finding routines and performing behavioural patterns that previously proved successful [52]. During reactive coping, animals attempt to modify their behaviour to find the best way to handle every new potential stressor [53].

Different coping styles are based on the differential use of various physiological and neuroendocrine mechanisms [30]. The general impression is that these mechanisms consistently vary in the same direction across species, showing higher HPA axis and sympathetic reactivity and lower parasympathetic reactivity in proactive animals, and the opposite in reactive animals [39].

Indeed, cattle are different in their personality and their coping styles. Personality traits seemed to be consistent over time [46]. Personality (temperamental or calm in a challenging situation, impulsive or reserved when approached by humans) is grounded by differences in autonomic activity in cows. The sympathetic tone was higher, while vagal activity was lower in temperamental cows than in calm animals during rest. The same difference in heart rate variability during rest was observed in the cows who reacted impulsively to the approach of humans [45]. Another study demonstrated that more reactive animals exhibit increased plasma and salivary cortisol concentrations and higher cardiac autonomic responsiveness to transrectal examination (acute pain) than less reactive cows [54].

## 3. Behavioural Reactions to the Different Origins of Stress

**Figure 1:**
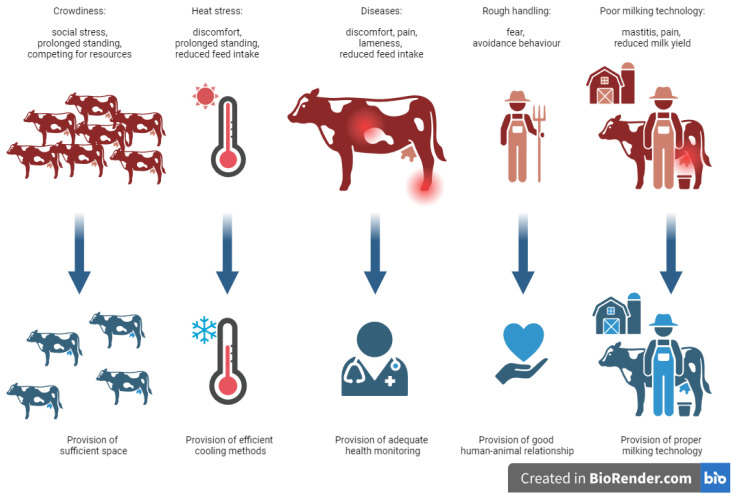
The most frequent stressors affecting the behaviour of dairy cattle, their main symptoms and possible solutions.

In dairy cattle farming, animals face several stressors related to housing technology and arising from the husbandry itself. These can be overcrowding, heat stress, pain caused by inflammation or diseases related to internal origins or severe lameness, the presence of the farmer, inappropriate human–cattle relationships, or milking as the most common short-term stressors in dairy cattle housing systems (Figure 1). Animals react to these stressors with behavioural responses, such as prolonged standing, reduced lying times, abnormal gait, decreased activity and rumination, or avoidance of humans. In the following, stress-inducing factors and the changes in the behaviour of dairy cattle are discussed.

### 3.1. Pain and Disease

Behaviour is traditionally included as a component of subjective assessment performed during clinical examinations; however, objective behaviour measures are less common [55].

Pain experienced in specific circumstances can induce a stress response when the animals are re-exposed to those circumstances [56]. Measures are often used to detect pain in animals’ behaviour. Several techniques can quantify cattle behaviour in the field, including frequency and duration of behavioural elements, such as vocalisation, arched back, or attention to the painful body area. Behavioural responses can provide an appropriate indication of the duration and different phases of a painful experience [55,57,58,59]. The behavioural effects of painful husbandry procedures causing tissue damage have been studied in dairy calves, focusing on the test of the efficacy of analgesics. Although studies demonstrate that pain-evoking husbandry procedures affect behaviour, reports suggest that pain caused by surgery can be reduced poorly by using local anaesthesia. Increased restlessness after ear-tagging was found in calves [60] similarly during the 5 min following hot-iron disbudding with and without local anaesthesia [61]. In another experiment, Stewart et al. [62] found no effect of local anaesthesia on dairy calves’ behavioural responses to pain caused by dehorning or castration. A more pronounced escape–avoidance reaction of the hot-iron-disbudded Angus calves was found compared to calves exposed to freeze disbudding, indicating a higher pain sensation perceived by hot-iron-branded animals [63].

After calving, sick cows tried to separate themselves and spent less time with the calves [64]. Ill cows housed in partially covered pens took less time with feeding, tended to spend more time lying down, and spent more time in the corner of the pen than healthy cows. In another study, cows who underwent dystocia showed depressed maternal behaviours after calving [54].

It is assumed that the increased activity around calving is due to pain. Deviation from normal daily activity patterns around calving (increased or reduced) indicates additional pain due to dystocia or a cow delivering a stillborn calf [65].

Primiparous cows with metritis spent more time lying down and had reduced activity, whereas multiparous cows reduced their number of lying bouts [66,67,68]. These activity pattern changes are possibly associated with sickness behaviour and avoidance of pain during lying movements [69].

In the case of mastitis, cows are more restless during milking, usually spend less time lying, change the lying position, and increase the frequency of lying bouts [70]. Others reported that cows spend more time ‘standing idle’ and less time feeding, ruminating, and self-grooming during the 24 hours after udder infection [71].

Besides mastitis, lameness is associated with significant pain and is thus considered to cause chronic stress [72,73], especially when painful lesions are present for at least two weeks [74]. Lame cows are lower in rank and last when driven to the milking parlour or moved from the pasture [75]. Lameness also changes behaviour, as non-lame cows presented higher levels of aggressive non-nutritive behaviours than sound ones, and more aggressive contacts were received by lame animals [76]. Weight distribution when standing [77] and weight shifting between legs [78] are also important behavioural indicators of pain caused by lameness.

Even so, subclinical problems can cause minor, not entirely visible behavioural changes. At the same time, these changes can be discovered with precision livestock farming (PLF) technologies. For instance, cows with subclinical ketosis showed lower average rumination time and rumination chews, drinking time, chews per minute, and chews per bolus [79,80]. Cows with subclinical acidosis show similar behavioural changes, namely decreasing eating time and chewing activity [81].

### 3.2. Interactions with Humans

Human–cattle interactions can be distinguished based on the quality and goal of the interaction into four broad categories: human presence, human approach, human contact, and restraint [82]. The positive or negative impacts of human–cattle interactions are often studied through the animal’s behavioural responses to handling or restraint [45,83]. The magnitude and type of behavioural reactions to humans provide information about what an animal perceives as stressful, painful, or generally aversive. Furthermore, they provide insights into how cattle vary in basal behavioural traits such as temperament, personality, or coping style [42,84,85].

**Figure 2:**
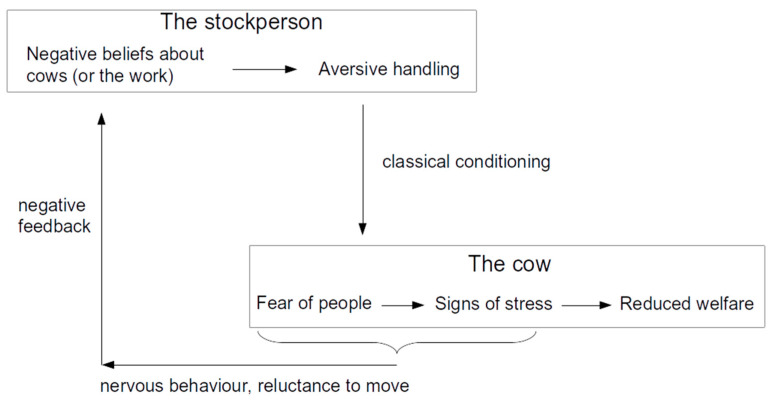
Human–cattle interactions concerning behaviour and welfare.

The stock person can most obviously affect the stress state of the animals through routine animal care tasks (Figure 2). Intensively managed dairy production involves several levels of interaction between humans and cattle. Most of these interactions are associated with regular observation of the animals. Thus, this type of interaction often involves only visual contact between the stock person and the animals. In addition to visual and auditory communication, stock people usually use tactile interactions to move cattle in most production systems. Human–cattle interactions also occur where animals must be restrained and subjected to management or health procedures.

The human–cattle relationship could be improved by gentle tactile contact [86,87], which is thought to mimic social licking, an affiliative behaviour shown by cows [88]. Gentle tactile stimulation, often combined with talking to animals in a gentle voice, reduces the avoidance distance of cows [89,90] and calves [91,92] and the fear of humans [93]. Brushing animals for seven consecutive days or passage through the working chute for seven consecutive days as habituation protocols also showed benefits in terms of the human–cattle relationship in dairy calves, and heifers had the most remarkable behavioural improvements [94]. Furthermore, these heifers responded more calmly during student–animal interactions in class. Therefore, the authors concluded that this was beneficial for the safety of the students and animals. In another study with dairy cows, interacting gently with animals in free-stall barns improved the human–animal relationship to a higher degree than interactions during restraint in the feeding rack [95].

There is scientific evidence that human–cattle relations are animal welfare-related questions and subjects of economic issues. Dairy farmers with a positive attitude towards animals and their everyday work are likelier to obtain better production results and higher milk production [96]. It was also shown that fearful cows produce less milk [97,98]. In contrast, others reported that the human–cattle relationship is associated with udder health [99].

Pajor et al. [100] evaluated the stimulation level through different rewarding/punishing methods, such as brushing, feeding, hitting/shouting, and other cow-driving practices, such as tail twisting, electric pods, and shouting. The authors demonstrated that feeding was a kind of motivation, that hitting/shouting triggered resistance, and that all the tested driving methods made the cows’ behaviour resistant. In another experiment, cows feared the aggressive handlers at the place of treatment and their barn. The animals could recognise the handlers and generalise their earlier experiences [101]. An interesting finding was that the colour of the clothes was important in recognition [102].

The avoidance distance is an appropriate indicator (concerning high inter-observer reliability and repeatability) of the human–cattle relationship [103], as it increased on farms where rough handling was found to be frequent [104,105,106]. Behavioural and cortisol responses of multiparous Holstein cows were compared in a study during two trial periods: (1) milked in a herringbone parlour and frequently experiencing aversive handling (shouting and sometimes hitting while moving cows to the milking parlour) and (2) two months after the introduction of a milking robot where human interaction was minimal [107]. Higher sympathetic activity and faecal glucocorticoid concentrations during the parlour milking period indicated that the automatic milking process was less stressful for the cows; however, the avoidance distance did not differ between the parlour and robotic milking periods. This suggests that cows remember their bad experiences with humans. A question can arise of how long these bad memories last in cattle. There is no evidence to answer this question. Positive handling had prolonged effects on the avoidance reaction for eight weeks [86], so negative experiences can be suspected to last at least this period.

### 3.3. Heat Stress

As homeothermic animals, dairy cows’ bodies can only function within a limited, relatively narrow temperature range. Internal body temperature increases due to physiological and biochemical processes (such as digestion and metabolism, muscular activity, etc.) and environmental heat [108]. By reducing daily dry matter intake, heat stress has numerous and wide-ranging adverse effects on cows. As a result, on the one hand, nutrient deficiency develops compared to needs. Therefore, milk loss, impaired fertility, ketosis, oxidative stress, and protein deficiency may occur, and mineral, microelement, and vitamin supply may also be deficient. As a result, immunosuppression and various diseases can develop [109]. On the other hand, fermentation processes in the rumen may also be disturbed, further aggravating diseases due to the lack of certain nutrients. One of the common concomitants of heat stress is rumen acidosis, which, in addition to deteriorating feed conversion, can even cause organic diseases [110]. Studies examined how heat stress affects cattle’s natural coping behaviours, as being under heat stress may change their behaviour to improve cooling [111]. Works have shown behavioural coping strategies including increased water intake, decreased feed intake, prolonged standing time, and decreased rumination [112,113,114]. There is a change in water intake patterns during heat stress, as cows increase drinking frequency, but they consume a lower quantity per visit [115]. Moreover, the cows spend more time at the drinker and engage in more competitive events for the water. Cows with low competitive success at drinkers shift their drinking behaviour to avoid the drinker during the hottest and most competitive time of day [116]. During heat stress episodes, cattle seek shade to protect against solar radiation. If no shade is available, they continue to stand to attempt to dissipate the accumulating heat [117]. As ambient temperature increases, dairy cows reduce lying time by 30% to increase body surface area for heat dissipation [118]. When exposed to mild to moderate heat stress, cows spent more time standing and decreased activity to increase body surface area [111,119].

Studies on adult cows indicate that extended heat stress increases activity and compromises rest time. The results further suggest that rest time can be a more appropriate parameter than activity to describe the effects of heat stress on lactating dairy cows [120]. Heat stress also causes changes in the frequency of lying down and the length of lying time in dairy calves [121]. In this study, additional shading reduced the frequency of lying down on days with a maximal temperature–humidity index above 78; however, it did not affect lying time.

### 3.4. Stress Caused by Technology

#### 3.4.1. Milking

Behavioural responses have been used to assess stress experienced by dairy cows during milking. Some authors found higher stress levels in robotic milking systems than in auto-tandem parlours [122,123]. In contrast, others found no such differences [124] or even observed lower degree of restlessness behaviours compared to a herringbone parlour [125]. Some studies proved that parity could also influence the expression of agonistic behaviours, like displacement, blocking, and hesitation, before entering the milking robots [126,127]. Tail swishing and displacement behaviour were more frequent in first-lactation heifers compared to multiparous cows [128]. Solano et al. [129] reported that primiparous cows had longer waiting times than multiparous cows before entering the milking stalls. Similarly, Dijkstra et al. [130] stated that first-lactation cows must wait longer to be milked due to the dominant structure. Another study reported that lower-ranking cows waited longer in the pre-milking holding pen than dominant cows before accessing the milking robot due to their lower social ranking [131]. The aforementioned behavioural responses are induced by the social dominance structure within the herd and are also seen when competing for other resources (e.g., feed, water, stall bed, brush).

An increased stepping rate indicated a sensitive period for animals after milking (after removing the last teat cup and before leaving the milking stall) in a parallel milking parlour with a non-voluntary exit [132]. In this study, cows showed less stepping during both udder preparation and milking than during waiting after milking. Carousel milking has a clear benefit regarding stress over parallel or herringbone parlours with stationary milking stalls and a side-opening design [133]. Although, like earlier findings [134], being in the holding pen is stressful for cows; vagal predominance, the low frequency of steps, and the high prevalence of rumination during milking suggest that due to the continuous visual contact between cows, the milking process is less stressful for the animals in rotary parlours than in stationary milking stalls [133]. Nevertheless, it should be noted that findings might be specific to the investigation farm as several factors would influence the behavioural responses of cows to milking, e.g., the experience of the animals [135] or stockmanship [136].

#### 3.4.2. Bad Comfort, Crowding and Social Stress

Several studies explain how floor types, tie-stall design [137], and bedding quality [138] affect comfort-related cow behaviours. In addition to total lying time, the environment affects other components of lying behaviour; these responses can explain why behavioural changes occur [139]. Schütz and Cox [140] evaluated the effects of short-term exposure to different flooring surfaces on the lying behaviour of dairy cows. They found that cows on wood chips spent the most time lying, and cows on concrete spent the least time lying compared with those on rubber mattresses with different thicknesses. Tucker et al. [141] showed that sawdust bedding increased lying time. It improved cow comfort in stalls with geotextile mattresses. In contrast, others demonstrated that bedding quality is essential for comfort, as, after =five weeks on a stand-off pad, cows lay down less [142].

Besides stall comfort, social factors such as stocking density and regrouping also significantly impact dairy cattle behaviour [143]. Higher density leads to shorter lying times, and animals spend more time outside the stalls. There are increasing displacements, and the animals try to rest earlier after milking [144]. A higher stocking density increases the number of aggressive non-nutritive behaviours at the feeding bunk and displacements and decreases feeding bouts. However, using a headlock or barrier [145] or feed stalls [146] reduces aggression, competition, and the average number of displacements at the feed bunk. Recent studies indicate that overstocking stalls and headlocks to about 115% did not affect daily lying and rumination time [147,148]. Increasing stocking density above 130% resulted in reduced daily lying time [144,148], decreased feeding time [149], and reduced the ratio of time spent ruminating [140]. When multiparous cows were examined in normal (1 box/cow) and crowded (0.5 box/cow) situations around parturition, longer standing times were observed at higher animal density, and there were more displacements at the feed bunk. Cows fed more frequently in shorter feeding bouts and decreased overall dry matter intake [149]. In a more recent study, Wang et al. [150] demonstrated that understocking supported the expression of cows’ natural behaviours, including lying, feeding, and rumination behaviour; however, in contrast to the studies mentioned above, overstocking did not harm behaviour and comfort indices.

Social dominance has an impact on the behaviour of the cows in a competitive situation. After fresh TMR delivery, high-ranking cows spent more time at the feeder compared with low-ranking cows [151], which could potentially alter digestion due to sorting by dominant cows [149,152]. Cows exposed to an unpredictable and competitive social environment changed how and when they consumed their diet and showed differences in some physiological biomarkers [153]. Regrouping is a frequent management method on dairy farms, having disruptive effects on behaviour and production [143], although the presence of a small group of familiar cows upon regrouping may provide social support and mitigate some of the negative effects [154].

## 4. Strategies for Managing and Reducing Stress in Dairy Cows

Among stressors, heat stress is the one that has been most extensively studied, and the most detailed recommendations are available for its management [111,155]. To mitigate the harmful effects of heat stress, it is primarily recommended to regulate the microclimate of livestock buildings and provide solutions that increase the efficiency of heat dissipation of animals, such as wetting the body surface and then blowing it with fans [156,157,158]. In addition, improving the efficiency of rumen function with feed additives (live yeast, probiotics like Direct Feed Microbials or DFMs, enzymes) and nutritional measures are useful [159,160,161]. An example of the latter is the proposal to shift feeding sessions in summer to cooler times of day when the dry matter intake is higher [112]. Total Mixed Ration (TMR) or Partially Mixed Ration (PMR) with a homogeneous structure can also significantly contribute to successful stress management by preventing dominant animals from selecting valuable components from the feed [162].

The primary prevention and treatment for social stressors is maintaining the correct stocking density and avoiding overcrowding. Stocking density is determined by the dimensions of the barns and pens based on the available capacity for feeding, drinking, and resting space [163]. It would also help if the farmer could ensure separate housing for first-lactating and multiparous cows [164]. Social stress can be reduced by maintaining well-sized and stable groups, which can be ensured through thoughtful, good grouping practices [165]. Less frequent grouping is recommended, and (if grouping is unavoidable) a few animals should be transferred to the new group at a time. Thus, the latest members of the group are dissipated by any aggressive behaviour of the old group members [166]. A predictable environment and social group help the cows cope with stressors [153].

Intelligent lighting systems that provide rest for cows and allow sufficient lighting to carry out human work in robotic barns with continuous operation have also been spreading. Constant, 24 h long lighting leads to the exhaustion of cows, significantly impairing their production capacity. Animals should generally be provided 16 h of light and 8 h of darkness. However, the 8 h dark period can also be lit with red light, which no longer interferes with the cows’ rest and allows them to leave the rest area to visit the milking robot, but humans can also do their job [167].

Environmental enrichment is not widespread in dairy cattle farming, but some elements are widely used. Such is the placement of scratching cow bushes in the barn, which animals are eager to visit [168]. Additionally, changes in the daily use pattern of brushes can be an early indicator of diseases like metritis [169].

In addition to the development of technological equipment, management also needs to change. Among the factors causing stress, overcrowding is of paramount importance. This can be addressed by putting it into practice and applying good management. During the transition period, it is crucial to avoid overcrowding. Therefore, livestock buildings should only be filled with animals up to 80% of their nominal capacity [170]. Not only the rest areas available in the barn can determine the stocking density, but also the access to the feeding space and drinkers. With the lowest access, the source above determines the number of animals that can be accommodated in the barn.

## 5. Conclusions

The first response of dairy cattle to stress is changing behaviour, which is an efficient way to cope with aversive situations. The literature reviewed demonstrates several stressors that affect dairy cattle in intensive housing systems, including pain, diseases, heat exposure, technology, and social interactions. Animals show a variety of behavioural responses to answer these challenges. Farm people and veterinarians need to recognise behavioural changes indicating possible stress on their animals. Early detection is the key to an effective intervention to decrease stress and help the animals cope. With increasingly popular sensor technology in PLF, animals’ acute behavioural stress responses can be effectively detected.

Even though stressors do not often occur unaccompanied and only once, and chronic stress consequential from facing numerous acute intermittent stressors is thus more frequent [171], scientific data regarding the acute behavioural reactions of chronically stressed cattle are still lacking. Therefore, research is still necessary concerning the questions of chronic stress and its interactions with acute stress expressed in changes in cattle behaviour. Automated measurement of feeding and lying behaviours would allow the detection and reduction of chronic stress, resulting in higher welfare for cows and revenues for farm practitioners. Based on the existing literature, temperament and coping style are recommended as important traits to be considered in assessing individual behavioural stress responsivity in cattle. As there is disagreement between producers and veterinarians about pain management [172], and attitudes towards the pain of cattle farmers are controversial [173], there is a need to study the behavioural reactions of cattle to pain for a better understanding of the relationships between pain and behaviour. Coping styles should also be investigated in these studies based on validated behaviour tests.

## Data Availability

The data presented in this study are available on request from the corresponding author.
